# Liver sinusoidal endothelial cells (LSECs) modifications in patients with chronic hepatitis C

**DOI:** 10.1038/s41598-019-45114-1

**Published:** 2019-06-19

**Authors:** Andrea Baiocchini, Franca Del Nonno, Chiara Taibi, Ubaldo Visco-Comandini, Gianpiero D’Offizi, Mauro Piacentini, Laura Falasca

**Affiliations:** 1 Pathology Unit, Department of Epidemiology and Preclinical Research, National Institute for Infectious Diseases Lazzaro Spallanzani-IRCCS, Rome, Italy; 2 Infectious Disease-Hepatology Unit, POIT Department, National Institute for Infectious Diseases Lazzaro Spallanzani-IRCCS, Rome, Italy; 3 Department of Biology, University of Rome “Tor Vergata”, Rome, Italy; 4 Laboratory of Electron Microscopy, Department of Epidemiology and Preclinical Research National Institute for Infectious Diseases Lazzaro Spallanzani-IRCCS, Rome, Italy

**Keywords:** Liver fibrosis, Hepatitis C

## Abstract

The sinusoidal endothelial cells present in the liver (LSECs) are tipically characterized by the presence of pores (fenestrae). During some pathological conditions LSECs undergo “capillarization”, a process characterized by loss of fenestrations and acquisition of a vascular phenotype. In chronic liver disease capillarization has been reported to precede the development of fibrosis. LSECs modification in the setting of HCV infection is currently poorly investigated. Considering that HCV accounts for important changes in hepatocytes and in view of the intimate connection between hepatocytes and LSECs, here we set out to study in great detail the LSECs modifications in individuals with HCV-dependent chronic hepatitis. Electron microscopy analysis, and evaluation of CD32, CD31 and caveolin-1 expression showed that in HCV infection LSECs display major morphological changes but maintain their phenotypical identity. Capillarization was observed only in cases at initial stages of fibrosis. Our findings showed that the severity of LSECs modifications appears to be correlated with hepatocytes damage and fibrosis stage providing novel insight in the pathogenesis of HCV-chronic hepatitis.

## Introduction

The hepatitis C virus (HCV) infection is the prevalent cause of chronic liver diseases. It is estimated that yearly more than 350,000 people die worldwide from HCV-related liver disease^1^. HCV infection causes tissue damage, secretion of pro-inflammatory cytokines, and oxidative stress that contribute to progressive fibrosis, cirrhosis, and the eventual progression to hepatocellular carcinoma^2^.

Changes of hepatic sinusoids are crucial in the pathogenesis of liver cirrhosis. Liver damage is associated with molecular changes that lead to morphological abnormalities of liver sinusoidal endothelial cells (LSECs)^3,4^. LSECs can be defined as a unique microvascular cell type that differs morphologically and functionally from capillary endothelial cells due to the presence of the typical fenestrations, clustered in sieve plates, and absence of the basement membrane^5^. In normal liver, LSECs act as a selectively barrier between the blood and liver parenchyma. The LSECs by means of expression of scavenging receptors, together with extremely effective endocytic capacity, in few minutes can remove and recycle materials from the circulation, such as viral particles, advanced glycation end products and lipids^6,7^. LSECs also act as important mediators of lymphocyte adhesion and migration across the sinusoidal endothelium into the parenchyma^8^. In addition, LSECs maintain hepatic stellate cells quiescence, preventing liver fibrosis development^9,10^.

The protective properties of LSECs are lost following liver injury, and associated angiogenesis and vasoconstriction are promoted^11^. By using various experimental models of hepatotoxicity fibrosis, it has been demonstrated that LSECs can produce different kind of pro-inflammaotry mediators^12,13^. Upon injury, LSECs become profoundly deregulated, and this dysfunction has been showed to be associated with the phenomenon of capilarization^14^. The term “capillarization” indicates the loss of the specialized phenotype of the liver LSECs and the acquisition of normal features of capillaries^15^. As a response to liver injury LSECs lose the fenestrations, form a continuous basal membrane, and acquire pro-inflammatory, and pro-fibrotic features^16^. Recent studies demonstrated that capillarization contributes to the development of fibrosis in patients with chronic liver diseases^17,18^. Nevertheless, studies on different etiologic factors indicated that chronic liver damage may lead to fibrosis and cirrhosis through distinct events^19^. Despite common pathophysiological aspects of chronic hepatitis, differences are emerging in the mechanisms, even when “similar” causes, such as hepatitis B and hepatitis C virus-related cirrhosis^19^. This study was aimed to investigate the sinusoidal endothelial cells modification occurring in HCV-infected patients to verify whether LSECs in these patients differ from those affected by non-HCV-induced liver fibrosis.

## Results

### Baseline clinical characteristics

Demographic and clinical features of our cohort of HCV patients are reported in Table 1. Of the 24 patients enrolled, 58.4% were female. Median age was 50.6 years. Fibrosis stage was S2 for 6 cases (25%), S3 for 2 cases (8.3%), S4 for 7 cases (29.2%), and S5-S6 for 9 cases (37.5%) (Table 1). The genotyping results showed that the majority (75%) of the patients were infected by genotype 1. The proportion for genotype 1 among patients with different stage of fibrosis showed an equal distribution (Table 2).

**Table 1:** Patients’ characteristics at baseline.

Number of Patients	HCV+	HCV−
	24	6
**Demography**		
Age* (years)	50.6 ± 8.5 (30–63)	54.3 ± 14.3 (29–66)
Men (%)	10 (41.6)	3 (50)
Women (%)	14 (58.4)	3 (50)
**Morphometry**		
Fibrosis Stage N (%)		
Ishak score 0–2	6 (25)	2 (33.4)
Ishak score 3–4	9 (37.5)	1 (16.6)
Ishak score 5–6	9 (37.5)	3 (50)

^*^Media + SD; years (range).

**Table 2:** Frequency of HCV genotypes in liver fibrosis stage.

	Fibrosis stage (Ishak grading)		
	S0–S2 (n = 6)	S3–S4 (n = 9)	S5–S6 (n = 9)
**Viral Genotype**			
1	6	5	7
3	/	3	1
4	/	1	1

As controls, six HCV-negative subjects were also enrolled in the study. There was no significant difference as regard to the age and sex between the different groups (Table 1). Comparisons of the clinical characteristics between HCV positive or negative patients revealed comparable distributions of fibrosis stage (Table 1).

### Ultrastructural analysis of HCV infected liver

**Figure 1:**
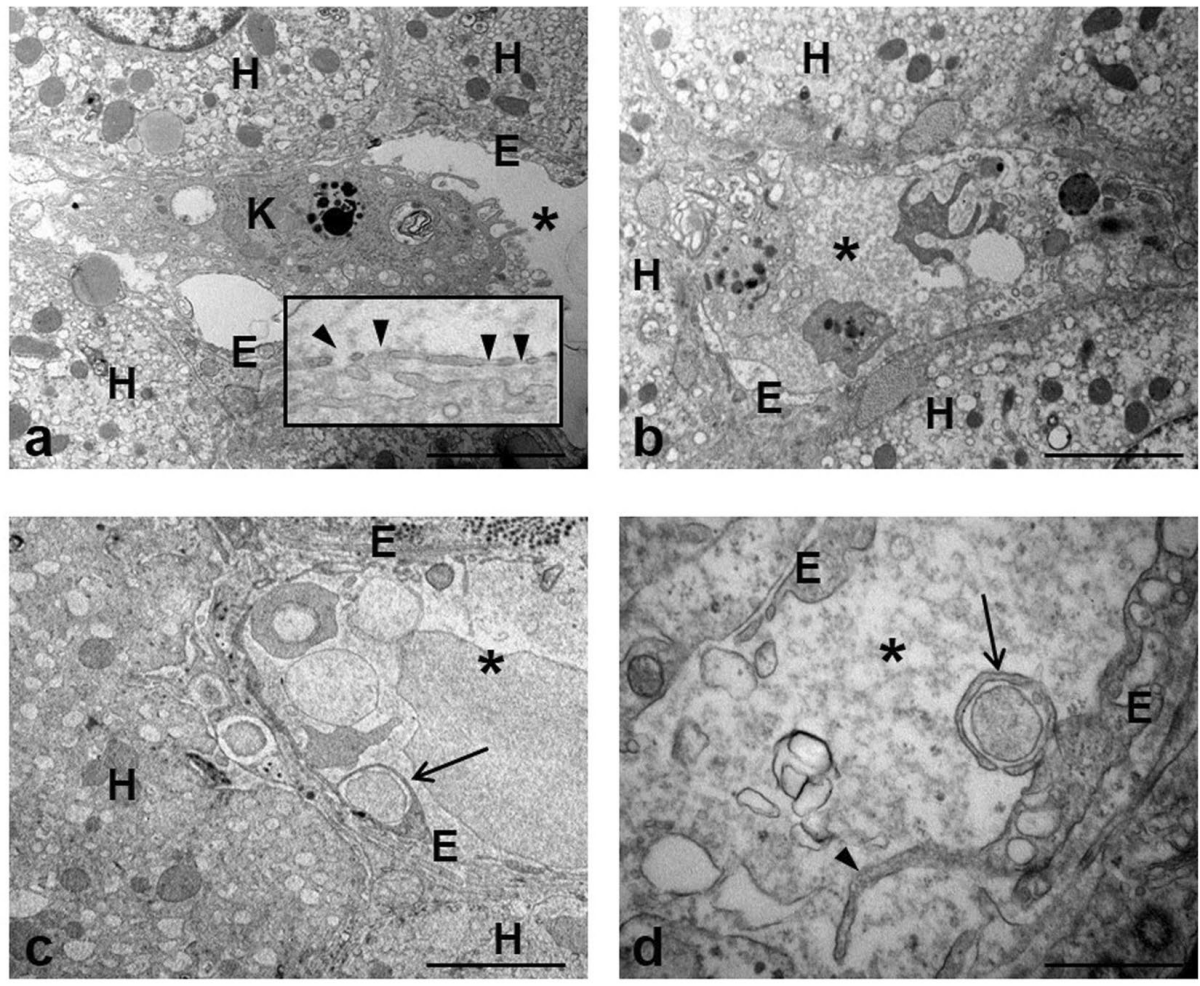
Transmission electron micrographs of human liver of chronically HCV-infected patient. (**a**) Representative image of a preserved liver sinusoidal endothelium (E) and neighboring hepatocytes (H); a Kupffer cell (K) is visible into the sinusoidal lumen (asterisk). The inset shows intact cytoplasmic processes of sinusoidal endothelial cells with typical fenestrae (arrowheads). (**b**) The image show the sinusoidal lumen (asterisk) crowded by the presence of inflammatory cells and material originating from damaged cells. (**c**,**d**) Microvilli (arrowhead) extending from the surface of endothelial cells (E) and the presence of engulfed material (arrows) inside the cytoplasm are visible. Scale bars: a, b, c = 5 μm; d = 1.2 μm.

Transmission electron microscopy was used to study the ultrastructural LSECs modifications occurring in the course of HCV infection. The typical morphological features of sinusoids, i.e. thin and fenestrated endothelium covering the space of Disse (Fig. 1a) were rarely observed. All liver samples from the HCV-infected patients showed different grades of cell injury, affecting both sinusoidal compartment and parenchyma. Sinusoidal lumen was frequently filled with amorphous material such as fibrin clot and cellular debris (Fig. 1b); this condition was often associated to unusual phagocytic capacity displayed by LSECs, demonstrated by the presence of long microvilli protruding at the cell surface that enclose particulate material, and by the observation of vacuoles with ingested material inside the cytoplasm (Fig. 1c,d).

**Figure 2:**
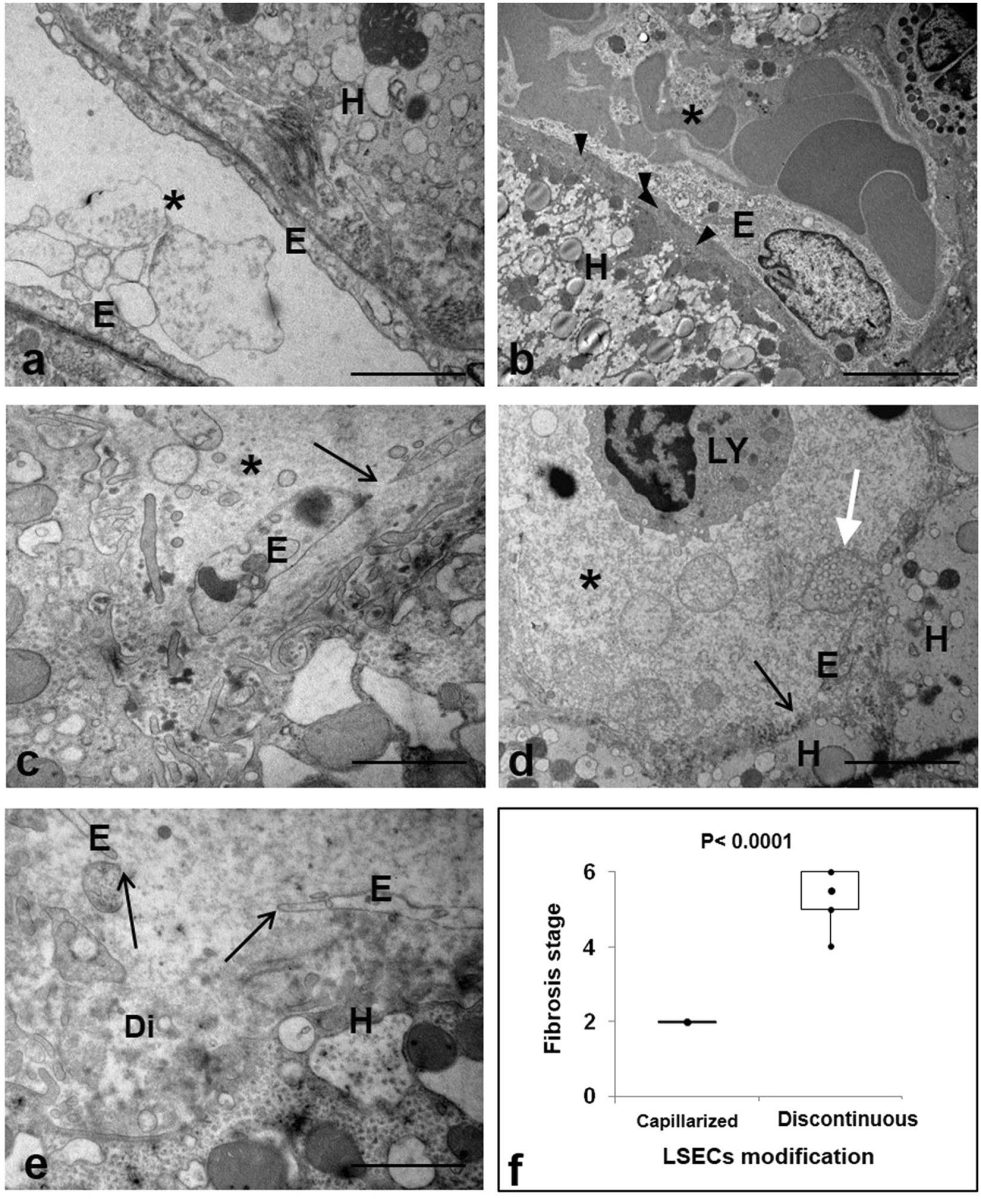
EM analysis of sinusoidal modifications in HCV-infected liver. (**a**,**b**) Representative electron micrographs of capillarized LSECs. The absence of fenestrae and the development of a continuous lining (**a**) with the appearance of abnormal basement membrane (arrowheads) on the basal side of LSECs is shown. (**c**) The micrograph shows the swelling of sinusoidal endothelium (E), and the presence of large gaps (arrows). (**d**,**e**) Images show variable degrees of discontinuous endothelial lining leading shedding of hepatocytes vesicles (white arrow) into the sinusoidal lumen (asterisk) and to a widening of the space of Disse (Di). (**f**) Box Plot representing the distribution of capillarized and discontinuous LSECs according with fibrosis stage (Ishak scoring system) in HCV-infected liver. (H) hepatocytes; (*) sinusoidal lumen. Scale bars: a,e = 1.2 μm; b–d = 5 μm.

**Figure 3:**
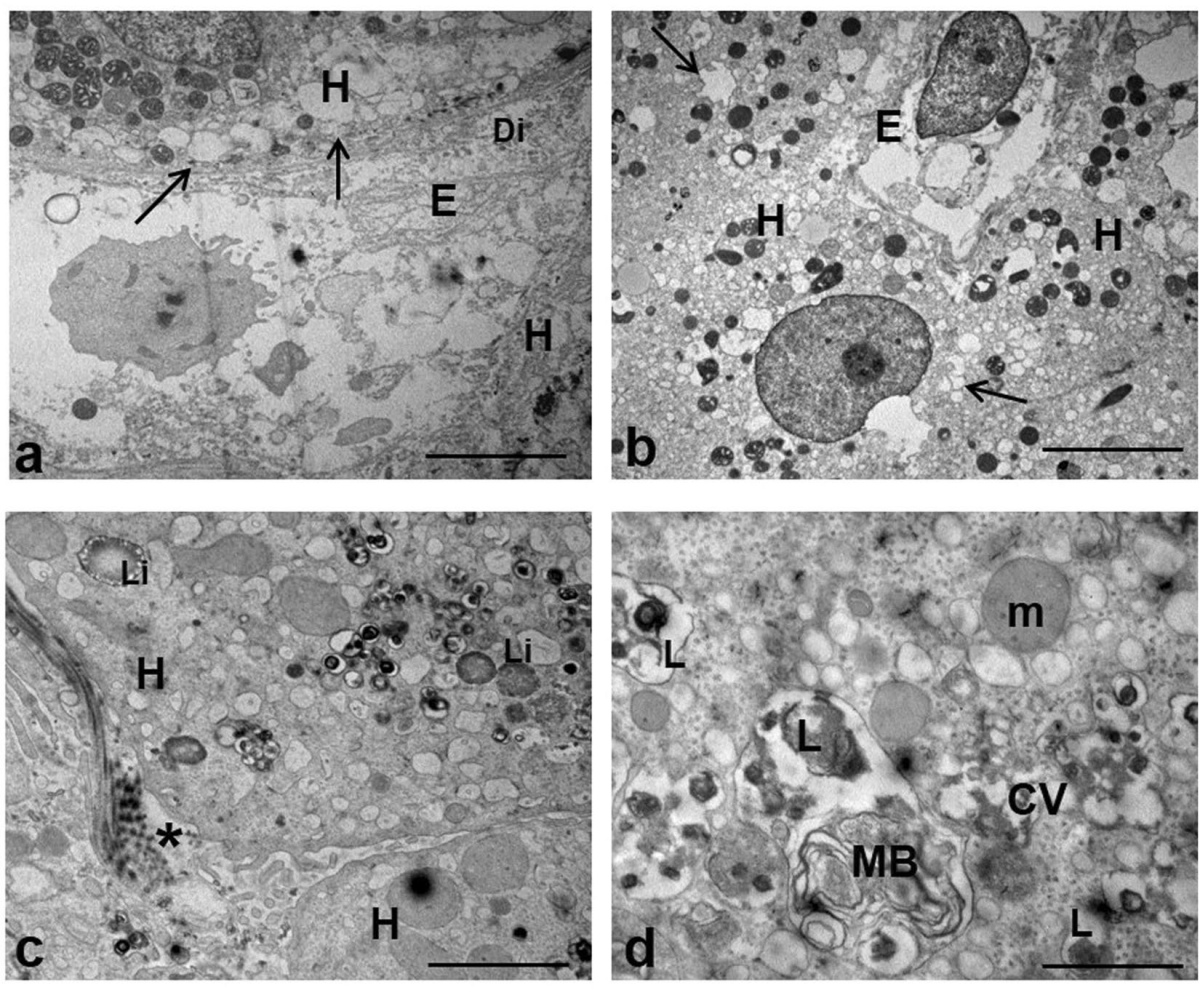
EM analysis of hepatocytes damage in HCV-infected liver. (**a**–**c**) Low-power overview showing parenchyma modifications induced by HCV, including the presence of extensive vacuolation (arrows), lipid droplets (Li), and perisinusoidal collagen (asterisk). (**d**) Higher magnification of a portion of hepatocyte cytoplasm with clusters of contiguous vesicles (CV), multilamellar body (MB), and lisosomal residual bodies (L). (H) hepatocyte); (Di) Disse’s space; (m) mitochondria. Scale bars: a, b = 5μm; c = 1.2 μm; d = 0.6 μm.

Of note, LSECs capillarization (Fig. 2a,b) was found in few cases (4/24) which were all diagnosed with S2 fibrosis stage (Fig. 2f; Supplementary Table S1). On the contrary, the analysis of the sinusoidal walls in liver samples from HCV-negative patients often showed the presence of continuous endothelial lining, associated with appearance of extracellular matrix deposition in Disse’s spaces resembling a basement membranes of vascular endothelium (Supplementary Fig S1). In the HCV infected livers LSECs showed various degrees of degeneration, ranging from cell swelling to deterioration and discontinuity in the endothelial lining, with the occurrence of gaps through which hepatocytes surface take direct contact with sinusoidal lumen (Fig. 2c–e). These latter dramatic alterations were observed in cases showing a fibrosis stage of S5-S6 (p ≤ 0.0001) (Fig. 2f). No correlation was found between the degree of LSECs alterations and virological and/or biochimical characteristics of the patients (Supplementary Table S1), while an association was observed with the extent of hepatocytes ultrastructural changes induced by HCV, consisting in endoplasmic reticulum dilatation, mitochondria abnormalities, and the presence of lipid inclusions, a large number of lysosomes, autophagosomes and lamellar bodies (Fig. 3).

### LSEC staining profile for CD32, CD31

**Figure 4:**
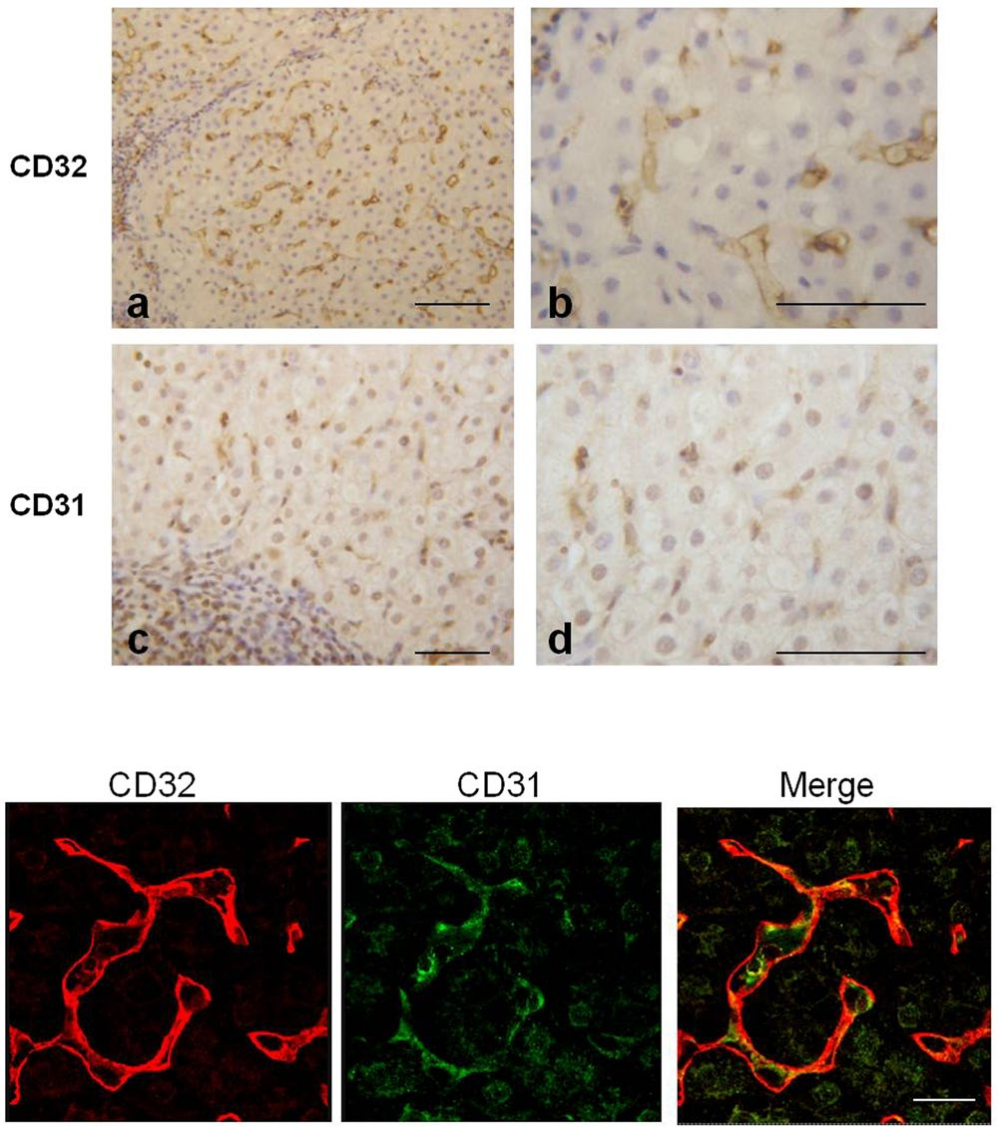
Expression of CD32 and CD31 in liver from HCV-infected. (Upper panel) Immunohistochemical analysis showed positive immunoreactions of both CD32 and CD31. (Lower panel) Confocal microscope images reveals a diversity in the localization of the two markers (CD32 is expressed at the cell surface; CD31 is present inside the cytoplasm) as demonstrated by the separated colors in the merged of the fluorescence signals. Scale bars: Upper panel a,c = 100 μm; b,d = 70 μm; Lower panel 25 μm.

Immunohistochemistry was used to characterize the LSECs phenotype. To this aim the expression of CD32, a protein marker of LSECs, and CD31, a marker of vascular endothelial cells, were assessed. Results obtained showed that expression of CD32 was not lost in HCV infected samples (Fig. 4a,b). Apparently in contrast with the positivity of CD32, the immunohistochemical staining for the vascular marker CD31 also showed a positive reaction (Fig. 4c,d). To better clarify whether the same cells co-expressed both markers, double immunofluorescence with CD32 and CD31 was carried out and analyzed under confocal laser scanning microscope. Results obtained showed that both markers were present on cells but with distinct sites of localization: CD32 was localized at the level of plasma membrane, while CD31 was visible inside the cytoplasm (Fig. 4). Interestingly it has been reported that in normal LSECs CD31 could have a cytoplasmic localization, whereas dedifferentiated/capillarised LSECs demonstrated expression of the protein at the cell surface^20^.

### Caveolin-1 expression

**Figure 5:**
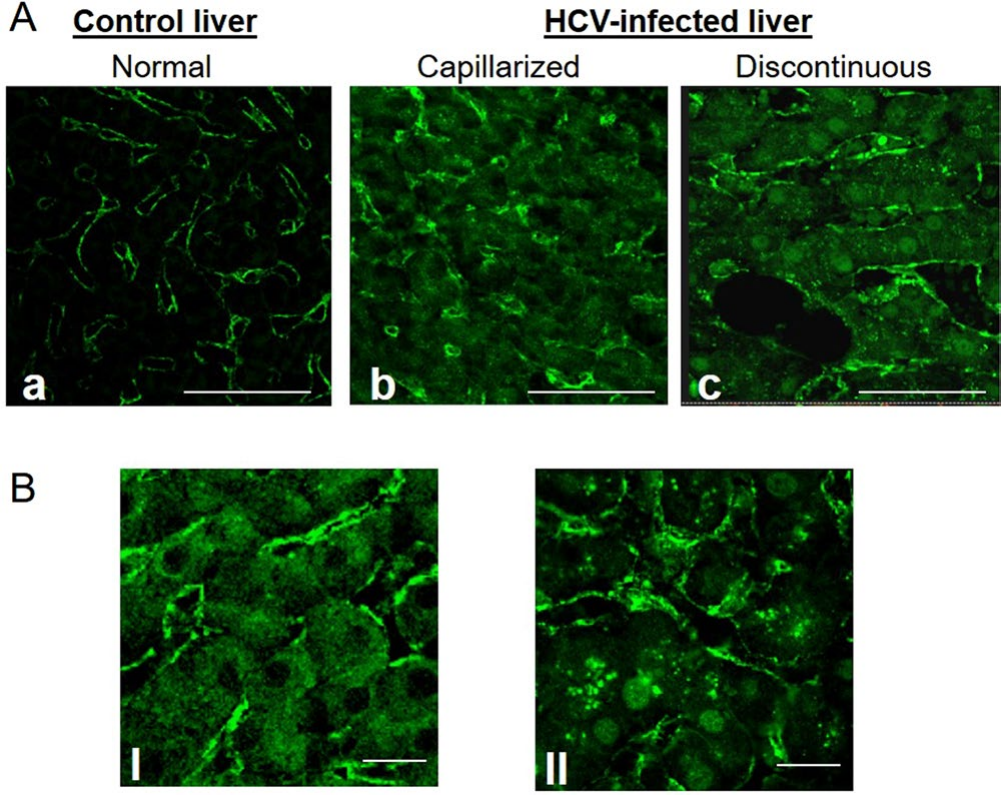
Confocal microscopy analysis of caveolin-1 (CAV-1) distribution. (Panel A) Normal liver (a) exhibits lack of CAV-1 expression in hepatocytes, while positive staining is restricted to the sinusoidal endothelial cells surface. In HCV-infected liver (b,c) the labeling is present both on hepatocytes and endothelial cells. (Panel B) Representative confocal images showing different staining pattern into parenchymal cells: the staining was found as absent in the nucleus and diffuse in the cytoplasm (I), or positive in the nucleus and present as puncta in the cytoplasm (II). Scale bar; A (a–c) = 25 μm; and B (I,II) = 12 μm.

**Figure 6:**
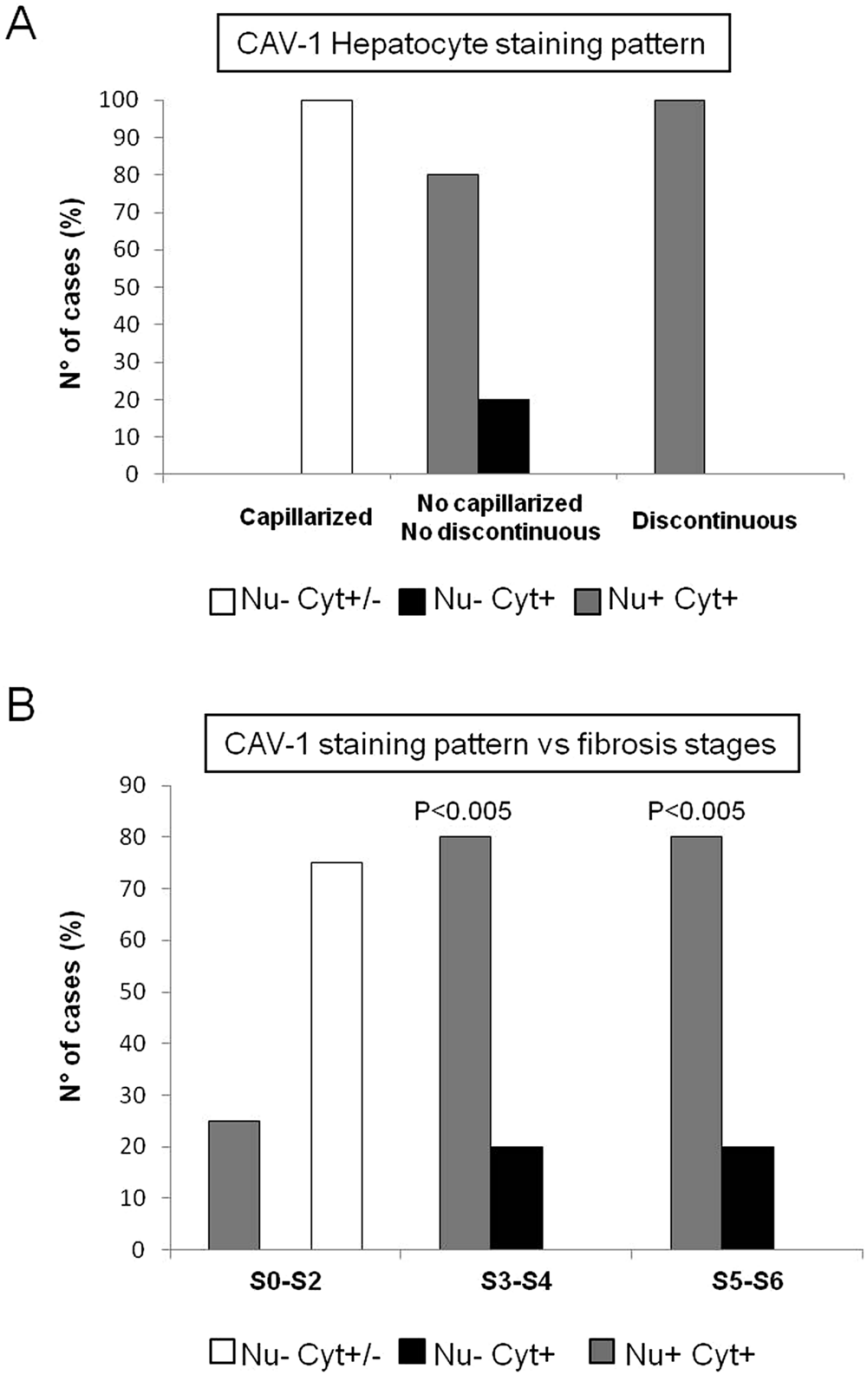
Distribution of CAV-1 staining pattern into hepatocytes. (Panel A) Distribution of the staining pattern in hepatocytes of HCV-infected liver according to the different LSECs modifications. (Panel B) Distribution of CAV-1 expression pattern according to fibrosis stages. Protein expression was analyzed by Chi-square test. A statistically significant association was found between CAV-1 nuclear expression and S3–S6 (P < 0.005; CI 95%).

Caveolin 1 (CAV-1), the principal scaffolding protein of caveolae, is present lining the walls of fenestrations^21^. Thus detailed localization of CAV-1 was analyzed by laser scanning confocal microscopy. In control liver the expression of CAV-1 was restricted to the sinusoidal walls, which exhibited a uniform and continuous labeling; no staining was observed at the parenchyma level (Fig. 5Aa). In the liver of HCV positive individuals LSECs maintained the positivity for CAV-1, even if the immunoreactivity was not regular along the endothelial layer, probably as a consequence of the morphological changes of these cells. Interestingly HCV-infected cases displayed positive reaction into hepatocytes. Different kinds of immunoreactivity pattern were found: the labeling was detected only in the cytoplasm or in both the cytoplasm and nucleus (Fig. 5A,B). In addition the staining was present as diffuse in the cytoplasm or concentrated in dots (Fig. 5B). We found that the different distribution of CAV-1 into hepatocytes correlated with LSECs modifications, in fact all cases presenting capillarization displayed absence of hepatocytes nuclear labeling, while 100% of cases in which LSECs appeared discontinuous displayed hepatocytes labeling not only in the cytoplasm but also in the nucleus (Fig. 6A; Supplementary Fig. S2). Analysis of CAV-1 expression demonstrated that the larger part of S0-S2 cases displayed absence of hepatocyte nuclear staining and faint cytoplasmic expression (Supplementary Fig. S3). Interestingly, a significant statistic association was detected between the nuclear expression and the fibrosis stage (P < 0.01) (Fig. 6B).

## Discussion

Microcirculation of the liver is unique among epithelial organs because it is not made by capillaries but by sinusoids. One of the peculiarity of the liver sinusoids is the presence a special endothelium characterized by the lack of a typical basement membrane and by the presence of fenestrations forming the so-called sieve plates^22^. A variety of agents and liver diseases influence the number and the size of fenestrae^7^. During fibrosis, there is a modification of LSECs that has been indicated as capillarization. This term indicate that these cells change their phenotype and become ordinary capillary, in particular, they lose the fenestrae peculiar of LSECs^23^. Although different opinions have been formulated on factors that influence LSECs modifications, the exact mechanism remains unknown and needs to be examined further. Most of the studied have investigated LSECs capillarization in humans alcoholic liver disease, or in experimental nonalcoholic fatty liver disease, as well as “*in vitro*” systems on isolated cells^16,24^. Both in animal models of cirrhosis and in humans after exposure to hepatotoxins, it has been shown that defenestration of the liver sinusoidal endothelium takes place at the beginning of the pathogenesis of cirrhosis^25^. Here we have focused on LSECs morphological alterations occurring in the setting of HCV infection. As resulted by electron microscopy analysis of liver samples at different stages of fibrosis, we provided evidences of a number of distinct ultrastructural abnormalities. We found the presence of long microvilli and large ingested vacuoles inside the cytoplasm; such features are typical of activated phenotype and differ from those of LSECs of normal liver, which are characterized by smooth surface, Considering the great amount of cell debris and material present into the sinusoidal lumen, which presumably saturated the scavenger capacity of Kupffer cells, the observation that LSECs may display phagocytic activity is not surprising, as also previously described^26^.

Interestingly we observed sinusoidal capillarization only in the S2 stage of HCV infected livers. It has been demonstrated that fibrosis-associated sinusoidal capillarization is aggravated by deletion of liver X receptor alfa (LXR*α*)^27^. Thus the absence of capillarization in our cases could be linked to the fact that both HCV core and NS5A proteins indirectly induce upregulation of LXR*α*^28^. It is very likely that capillarization of LSECs occurs with different dynamics in a variety of liver pathologies and it probably represents one among different kind of changes occurring in sinusoids during chronic liver diseases^29^. We also observed cell swelling and presence of large gaps (which appeared as a discontinuity in the sinusoidal wall). However, although the LSECs undergo these profound changes they maintain the peculiar phenotypic markers. Under physiological conditions sinusoidal endothelial cells express specific markers, such as CD32^30–32^. Our data showed that the presence of CD32 was maintained in livers of HCV patients, thus providing evidence of morphological abnormalities of liver sinusoidal endothelium in the presence of phenotypically normal LSECs.

The severity of changes we observed does not correlate with patients serum levels of HCV RNA, but appears to be associated to hepatocytes structural lesions and fibrosis stage. It can be hypothesized that the LSECs modifications, in HCV infected liver, could be dependent on the amount of virus into the hepatocytes^33^. Indeed, the crosstalk with the closely juxtaposed hepatocytes is of fundamental importance to preserve the hepatic sinusoidal endothelial cells differentiation. Since hepatocytes and endothelium do not establish direct cell contacts, this modulation is thought to be exerted by secreted factors^34^. Data from a prior report on isolated primary liver endothelial cells showed that LSECs are sensed by virus replication into hepatocytes and are able to produce an autocrine interferon signaling^35^.

Sinusoidal endothelial cells abundantly express caveolin-1, the major component of the caveolae structure. Cav-1 was originally identified lining the walls of fenestrations^21^. Currently, the expression of Cav-1 in HCV related liver pathogenesis remains largely unknown, even though an overexpression of Cav-1 in liver sinusoidal endothelial cells has been reported in late-stage cirrhosis^36^. However, this observation could be due to the fact that the Cav-1 expression which is one of the main cholesterol-binding proteins, can be induced in the cirrhotic liver due to higher levels of cholesterol^37^. In this study, for the first time, we demonstrate a pattern of distribution of CAV-1 into hepatocytes of HCV- infected patients which differs from uninfected livers. Our results show a specific localization of caveolin in the nucleus and in cytoplasmic compartments. The nuclear localization of Cav-1 has been reported to be involved in gene transcription regulation^38^, and a role of CAV1 in autophagy induction has been reported^39^. In this regard it is interesting to note that HCV controls and modifies the biogenesis of autophagosomes for its own replication^40^.

In conclusion, we showed in our cohort of HCV infected patients, that LSECs undergo morphological changes associated to hepatocytes damage. Our results provide a further confirm of the idea previously reported^41^, that LSECs are sensitive to HCV replication in hepatocytes and can actively respond playing a defensive role. A larger number of patients should be analyzed to further enhance the significance of our findings, however this study has the merit of being the first to provide detailed ultrastructural evidences of what happens in the sinusoidal compartment of the liver as a consequence of HCV chronic infection. New insights in HCV induced liver injury, can help to design alternative therapeutic strategies upon removal of the causative agent, in the era of virus eradication with direct-acting antivirals (DAAs).

## Methods

### Patients and sample collection

24 consecutive patients diagnosed as infected by HCV, from the Infectious and Liver Diseases Unit at National Institute for Infectious Diseases (INMI) “Lazzaro Spallanzani” in Rome, Italy, were enrolled for this study. Partecipants to this study were included according to the following criteria: chronic HCV hepatitis assessed by liver biopsy and persistence of viral RNA in serum. Exclusion criteria were: HBV or HIV positivity, and use of alcohol or recreational drugs. Hemophiliac patients or individuals with other liver diseases were also excluded. Liver samples were obtained by liver biopsy performed on clinical indications for staging the liver injury. Peripheral blood samples were collected from patients for routine laboratory markers and biomarkers.

All enrolled patients in the study provided written informed consent to the research use of their biological samples, according to the Declaration of Helsinki principles. The study was approved by the Institutional Ethic Board (IEB) of the National Institute for Infectious Diseases “L. Spallanzani” (approval number: 41/2013). All data have been made anonymous to protect the confidentiality of participants.

Furthermore six, HCV negative liver specimens were obtained from retrospective cases within the local pathology archive (i.e. liver biopsy not selected for the purpose of the study). Replicate blocks were utilized, no longer required to be maintained on records. Cases considered included 2 primary sclerosing cholangitis, and 4 autoimmune hepatitis Presence of hepatitis B and/or human immunodeficiency virus, or alcoholic liver disease were considered exclusion criteria.

### Histological analyses

Liver biopsies were fixed in 10% neutral-buffered formalin, and paraffin-embedded. Sections of liver (4 μm) were stained with hematoxylin and eosin (H&E) and with Masson’s trichome for collagen fibers for assessment of liver fibrosis. For each biopsy sample was evaluated the histological activity index (HAI) and fibrosis according to the Ishak grading system for disease stage and grade^42^.

### Transmission electron microscopy analysis

Liver samples were processed for electron microscopy as previously described^43^. Specimens were fixed with 2.5% glutaraldehyde (Assing Spa) in 0.1 M cacodylate buffer for 1 h at 4 °C, and postfixed in 1% osmium tetroxide (Sigma-Aldrich) in 0.1 M cacodylate buffer. Samples were then dehydrated in graded ethanol and embedded in Epon resin. Thin sections were observed under a Zeiss EM900 transmission electron microscope. Images were captured with a digital camera Mega View II (SIS, Soft Imaging System GmbH, Munster, Germany).

To study LSECs ultrastructural morphology, liver blocks from all enrolled patients were analyzed by transmission electron microscopy. For each patient, five to six different non-overlapping areas containing sinusoidal spaces were randomly selected and investigated at different electron magnifications (ranging from 7000x to 50000x). At least ten micrographs per sample were captured. Representative images were shown in all Figures.

### Immunohistochemistry and immunofluorescence

Deparaffinized and rehydrated sections were used for immunohistochemistry, as previously described^44^. Liver sections were immersed in 10 mM sodium citrate, pH 6.0, and microwaved for antigen retrieval. After incubation with rabbit anti-CD31 (Novus) (1:100) and rabbit anti-CD32 (Abcam), 1:100 primary antibodies, a streptavidin–biotin-immunoperoxidase system with DAB (Biogenex, San Ramon, CA) as chromogen substrates was used to visualize positive reaction^45^.

For immunofluorescence, liver biopsy sections were incubated with rabbit anti-CD31 (Novus) (1:100), rabbit anti-CD32 (Abcam), 1:100, and rabbit anti-caveolin-1 (Santa Cruz) 1:100 primary antibodies overnight at 4 °C. Samples were then incubated with Alexa Fluor 488 or Alexa Fluor 594 conjugated goat anti-rabbit secondary antibodies for 1 h at 37 °C.

Analysis of fluorescence was performed with a TCS SP2 confocal laser-scanning microscope (Leica Microsystems, Wetzlar, Germany). Digital images using a 63X objective (zoom factor 2X) were obtained separately in both channels and acquired with Leica Confocal Software (LCS)^43^.

The quantitative assessment of caveolin-1 distribution was performed analyzing 5 cases per class of patients grouped according to the fibrosis stage (S0–S2, S3–S4, S5–S6). Evaluation of immune-positivity distribution was made in blind using at least five fields per sample (63 × objective). Representative images were shown in all Figures.

### Statistical analysis

Data were analysed using Prism GraphPad 5.0 software. Non-parametric, two-tailed, one-way ANOVA test was performed. Results with a P value of 0.05 or lower were considered statistically significant. To test for a potential association between study variables the Chi-square test was used.

## Supplementary information

Supplementary informations

## Data Availability

The datasets generated and/or analysed during the current study are available from the corresponding author on reasonable request.

## References

[1] Mohd Hanafiah K, Groeger J, Flaxman AD, Wiersma ST (2013). Global epidemiology of hepatitis C virus infection: new estimates of age-specific antibody to HCV seroprevalence. Hepatology.

[2] Kim A, Hepatitis C (2016). Virus. Ann. Intern. Med..

[3] Iwakiri Y, Shah V, Rockey DC (2014). Vascular pathobiology in chronic liver disease and cirrhosis—current status and future directions. J. Hepatol..

[4] Greuter T, Shah VH (2016). Hepatic sinusoids in liver injury, inflammation, and fibrosis: new pathophysiological insights. J. Gastroenterol..

[5] Wisse E, De Zanger RB, Charles K, Van Der Smissen P, McCuskey RS (1985). The liver sieve: considerations concerning the structure and function of endothelial fenestrae, the sinusoidal wall and the space of Disse. Hepatology.

[6] Poisson J (2017). Liver sinusoidal endothelial cells: Physiology and role in liver diseases. J. Hepatol.

[7] Shetty S, Lalor PF, Adams DH (2018). Liver sinusoidal endothelial cells — gatekeepers of hepatic immunity. Nat Rev Gastroenterol Hepatol..

[8] Shetty S, Lalor PF, Adams DH (2008). Lymphocyte recruitment to the liver: molecular insights into the pathogenesis of liver injury and hepatitis. Toxicology.

[9] Xie G (2012). Role of differentiation of liver sinusoidal endothelial cells in progression and regression of hepatic fibrosis in rats. Gastroenterology.

[10] Deleve LD, Wang X, Guo Y (2008). Sinusoidal endothelial cells prevent rat stellate cell activation and promote reversion to quiescence. Hepatology.

[11] Fan CQ, Crawford JM (2014). Sinusoidal obstruction syndrome (hepatic veno-occlusive disease). J. Clin. Exp. Hepatol..

[12] Connolly MK (2010). In hepatic fibrosis, liver sinusoidal endothelial cells acquire enhanced immunogenicity. J. Immunol..

[13] McMahan RH (2016). Free Fatty Acids Differentially Downregulate Chemokines in Liver Sinusoidal Endothelial Cells: Insights into Non-Alcoholic Fatty Liver Disease. Plos One.

[14] Farrell GC, Teoh NC, McCuskey RS (2008). Hepatic microcirculation in fatty liver disease. Anat. Rec. (Hoboken).

[15] Schaffner F, Poper H (1963). Capillarization of hepatic sinusoids in man. Gastroenterology.

[16] Pasarin M (2012). Sinusoidal endothelial dysfunction precedes inflammation and fibrosis in a model of NAFLD. PLoS One.

[17] Greuter T, Shah VH (2016). Hepatic sinusoids in liver injury, inflam-mation, and fibrosis: new pathophysiological insights. J Gastroenterol..

[18] DeLeve LD (2015). Liver sinusoidal endothelial cells in hepatic fibrosis. Hepatology.

[19] Karidis NP, Delladetsima I, Theocharis S (2015). Hepatocyte Turnover in Chronic HCV-Induced Liver Injury and Cirrhosis. Gastroenterol. Res. Pract..

[20] Lalor PF, Lai WK, Curbishley SM, Shetty S, Adams DH (2006). Human hepatic sinusoidal endothelial cells can be distinguished by expression of phenotypic markers related to their specialised functions *in vivo*. World J. Gastroenterol..

[21] Ogi M (2003). Distribution and localization of caveolin-1 in sinusoidal cells in rat liver. Med. Electron Microsc..

[22] Wisse E (1996). Structure and function of sinusoidal lining cells in the liver. Toxicol. Pathol..

[23] Le Couteur DG (2001). Pseudocapillarization and associated energy limitation in the aged rat liver. Hepatology.

[24] DeLeve LD, Maretti-Mira AC (2017). Liver Sinusoidal Endothelial Cell: An Update. Semin. Liver Dis..

[25] Braet F, Wisse E (2002). Structural and functional aspects of liver sinusoidal endothelial cell fenestrae: a review. Comp. Hepatol..

[26] Steffan AM, Gendrault JL, McCuskey RS, McCuskey PA, Kirn A (1986). Phagocytosis, an unrecognized property of murine endothelial liver cells. Hepatology.

[27] Xing Y, Zhao T, Gao X, Wu Y (2016). Liver X receptor α is essential for the capillarization of liver sinusoidal endothelial cells in liver injury. Sci. Rep..

[28] García-Mediavilla MV (2012). Liver X receptor α-mediated regulation of lipogenesis by core and NS5A proteins contributes to HCV-induced liver steatosis and HCV replication. Lab. Invest..

[29] Thabut D, Shah V (2010). Intrahepatic angiogenesis and sinusoidal remodeling in chronic liver disease: new targets for the treatment of portal hypertension?. J. Hepatol..

[30] Scoazec JY, Feldmann G (1991). *In situ* immunophenotyping study of endothelial cells of the human hepatic sinusoid: results and functional implications. Hepatology.

[31] Bioulac-Sage P (2010). Identification of liver sinusoidal endothelial cells in the human liver. Liver Int..

[32] Amarapurkar AD, Amarapurkar DN, Vibhav PND (2007). Angiogenesis in chronic liver disease. Ann. Hepatol..

[33] Xu Z (2017). Hepatitis C virus load in parenchyma cells correlates with hepatic injury in infected patients. Exp. Ther. Med..

[34] Natarajan V, Harris EN, Kidambi S (2017). SECs (Sinusoidal Endothelial Cells), Liver Microenvironment, and Fibrosis. Biomed Res. Int..

[35] Giugliano S (2015). Hepatitis C virus infection induces autocrine interferon signaling by human liver endothelial cells and release of exosomes, which inhibits viral replication. Gastroenterology.

[36] Yamazaki H (2013). Relation between ultrastructural localization, changes in caveolin-1, and capillarization of liver sinusoidal endothelial cells in human hepatitis C-related cirrhotic liver. J. Histochem. Cytochem..

[37] Shihata WA, Putra MRA, Chin-Dusting JPF (2017). Is There a Potential Therapeutic Role for Caveolin-1 in Fibrosis?. Front Pharmacol..

[38] Li W (2012). Caveolin-1 inhibits expression of antioxidant enzymes through direct interaction with nuclear erythroid 2 p45-related factor-2 (Nrf2). J. Biol. Chem..

[39] Nah J (2017). Phosphorylated CAV1 activates autophagy through an interaction with BECN1 under oxidative stress. Cell Death Dis..

[40] Wang L, Ou JJ (2018). Regulation of Autophagy by Hepatitis C Virus for Its Replication. DNA Cell Biol..

[41] Klenerman P, Ramamurthy N (2015). Liver sinusoidal endothelial cells: an antiviral “defendothelium”. Gastroenterology.

[42] Ishak K (1995). Histological grading and staging of chronic hepatitis. J. Hepatol..

[43] Piacentini M (2014). Characterization of distinct sub-cellular location of transglutaminase type II: changes in intracellular distribution in physiological and pathological states. Cell Tissue Res..

[44] Kowalik MA (2016). Induction of autophagy promotes the growth of early preneoplastic rat liver nodules. Oncotarget.

[45] Piacentini M (2018). Non-alcoholic fatty liver disease severity is modulated by transglutaminase type 2. Cell Death Dis..

