# Publisher Correction: Liver sinusoidal endothelial cells (LSECs) modifications in patients with chronic hepatitis C

**DOI:** 10.1038/s41598-020-58227-9

**Published:** 2020-01-24

**Authors:** Andrea Baiocchini, Franca Del Nonno, Chiara Taibi, Ubaldo Visco-Comandini, Gianpiero D’Offizi, Mauro Piacentini, Laura Falasca

**Affiliations:** 1 Pathology Unit, Department of epidemiology and Preclinical Research, National Institute for Infectious Diseases Lazzaro Spallanzani-IRCCS, Rome, Italy; 2 Infectious Disease-Hepatology Unit, POIT Department, National Institute for Infectious Diseases Lazzaro Spallanzani-IRCCS, Rome, Italy; 3 Department of Biology, University of Rome “Tor Vergata”, Rome, Italy; 4 Laboratory of Electron Microscopy, Department of Epidemiology and Preclinical Research National Institute for Infectious Diseases Lazzaro Spallanzani-IRCCS, Rome, Italy

Correction to: *Scientific Reports* 10.1038/s41598-019-45114-1, published online 19 June 2019

In the original version of this Article, the author Franca Del Nonno was incorrectly indexed. This error has now been corrected.

