# Supplementary material for: Liver sinusoidal endothelial cells (LSECs) modifications in patients with chronic hepatitis C
Source: Sci Rep. 2019 Jun 19;9:8760. doi: 10.1038/s41598-019-45114-1 (PMC6584733; doi:10.1038/s41598-019-45114-1)
Supplement: Supplementary file 1 — Supplementary informations [file 41598_2019_45114_MOESM1_ESM.pdf]

Supplementary Data

“Liver sinusoidal endothelial cells (LSECs) modifications in patients with chronic hepatitis C.”

Andrea Baiocchini, Franca Del Nonno, Chiara Taibi, Ubaldo Visco-Comandini, Gianpiero D'Offizi, Mauro Piacentini, Laura Falasca

Supplementary Figure S1

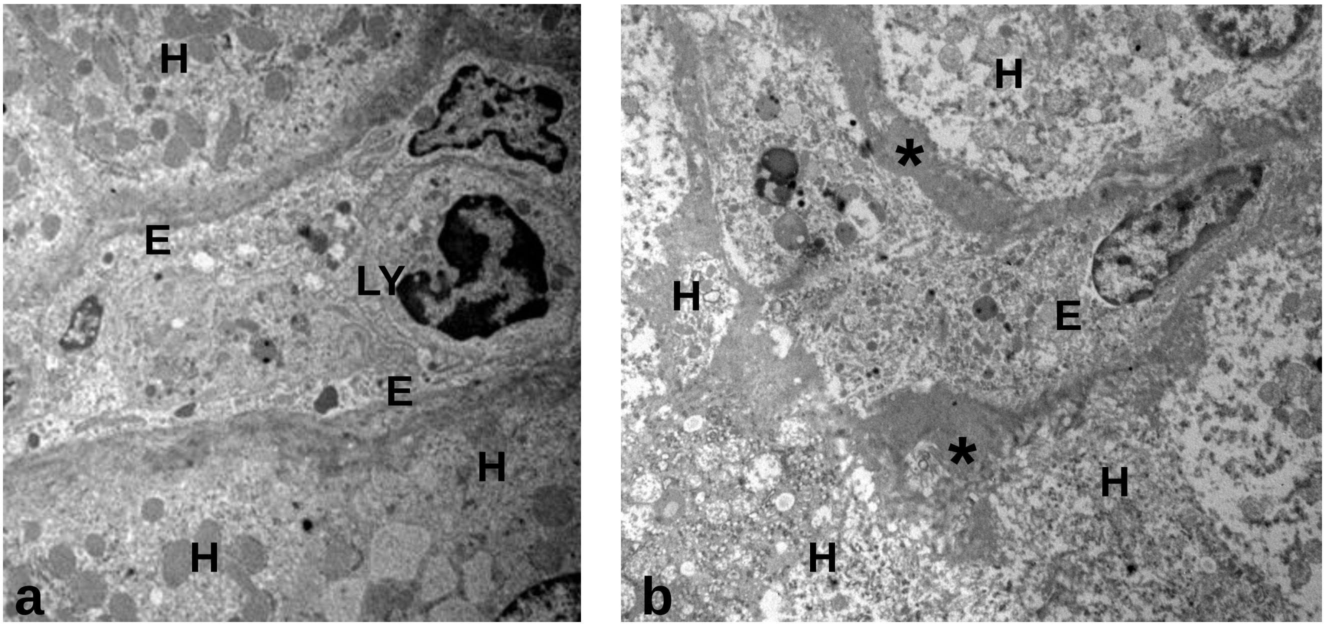

Representative transmission EM images of LSECs capillarization in liver from HCV-negative patients at early (a) and higher (b) stage of fibrosis.
A continuous endothelial cells (E) lining is associated to a basement membrane formation (*).

Supplementary Table S1

Pz (n.)	HCV genotype	HCV viral load (IU/mL)	Total cholesterol (mg/dl)	ALT (mg/dl)	AST (mg/dl)	Fibrosis stage	Capillarized LSECs (Yes/No)
1	1	22.9×10^5	150	353	262	S4	NO
2	1	46.7×10^5	150	75	51	S4	NO
3	1	2.9×10^5	178	16	21	S2	**YES**
4	1	56.9×10^5	230	68	43	S6	NO
5	3	41.2×10^5	82	183	122	S5	NO
6	1	4.44×10^5	111	109	153	S5	NO
7	1	3.01×10^5	142	39	33	S4	NO
8	1	1.2×10^5	210	44	32	S2	NO
9	1	1.4×10^5	119	35	31	S2	**YES**
10	1	0.6×10^5	182	52	42	S2	**YES**
11	1	4.96×10^5	210	104	95	S5	NO
12	3	0.2×10^5	124	50	46	S4	NO
13	1	3.4×10^5	168	43	52	S4	NO
14	1	1.3×10^5	150	103	81	S5	NO
15	1	33.9×10^5	181	65	102	S5	NO
16	1	2.2×10^5	166	25	33	S2	**YES**
17	4	12.2×10^5	172	36	41	S6	NO
18	1	15.4×10^5	152	65	53	S5	NO
19	3	0.7×10^5	164	84	143	S4	NO
20	3	33×10^5	134	34	26	S3	NO
21	1	$44,7 \times 10^5$	147	37	48	S3	NO
22	1	73.1×10^5	203	46	40	S2	NO
23	4	2.1×10^5	171	94	74	S4	NO
24	1	15.4×10^5	158	121	124	S6	NO

Supplementary Figure S2

Nucleus- cytop +/-

Patient | Ishak score

9 — S2

3 — S2

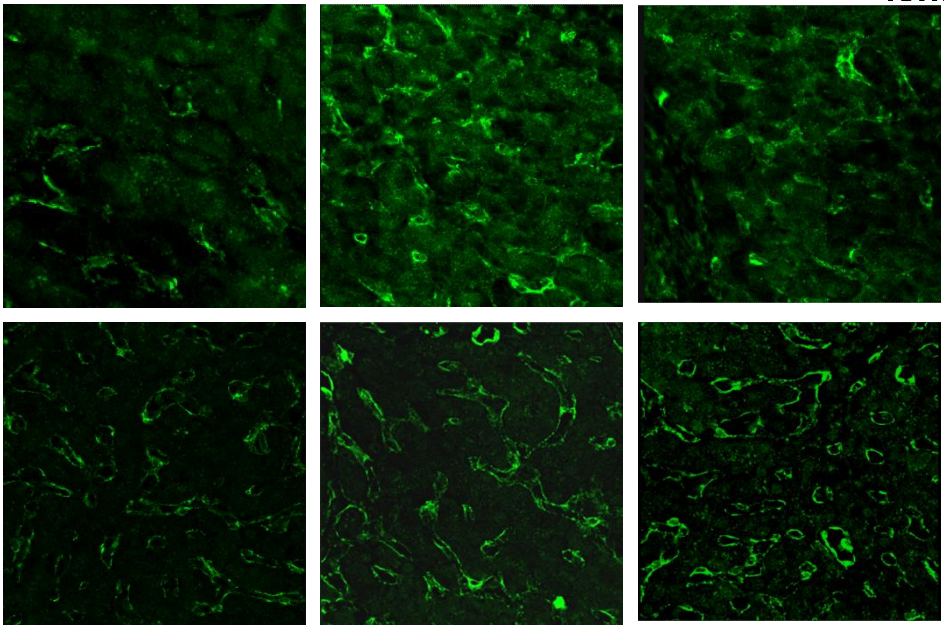

Capillarized

Nucleus+ cytop +

Patient | Ishak score

2 — S4

11 — S5

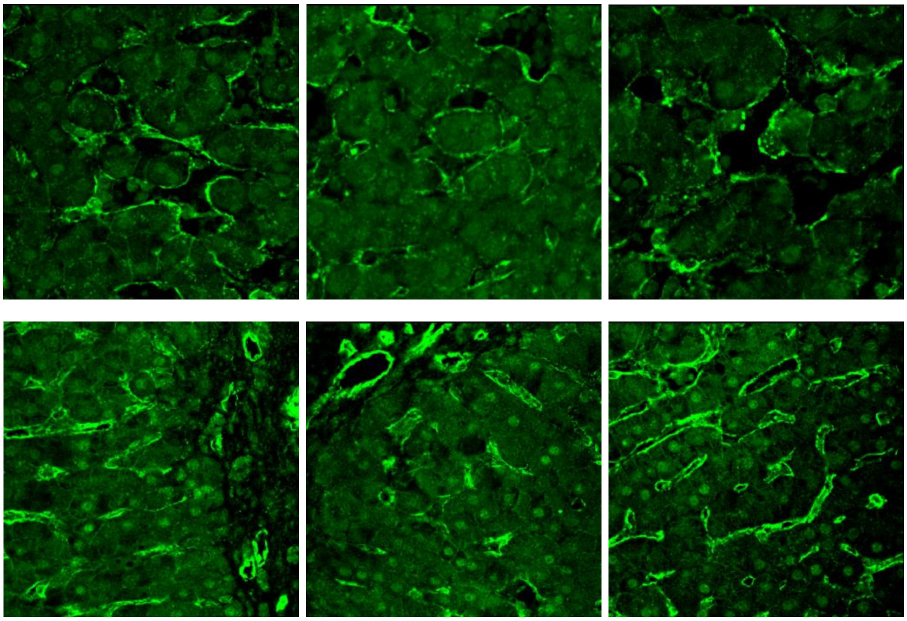

Discontinuous

Supplementary Figure S2

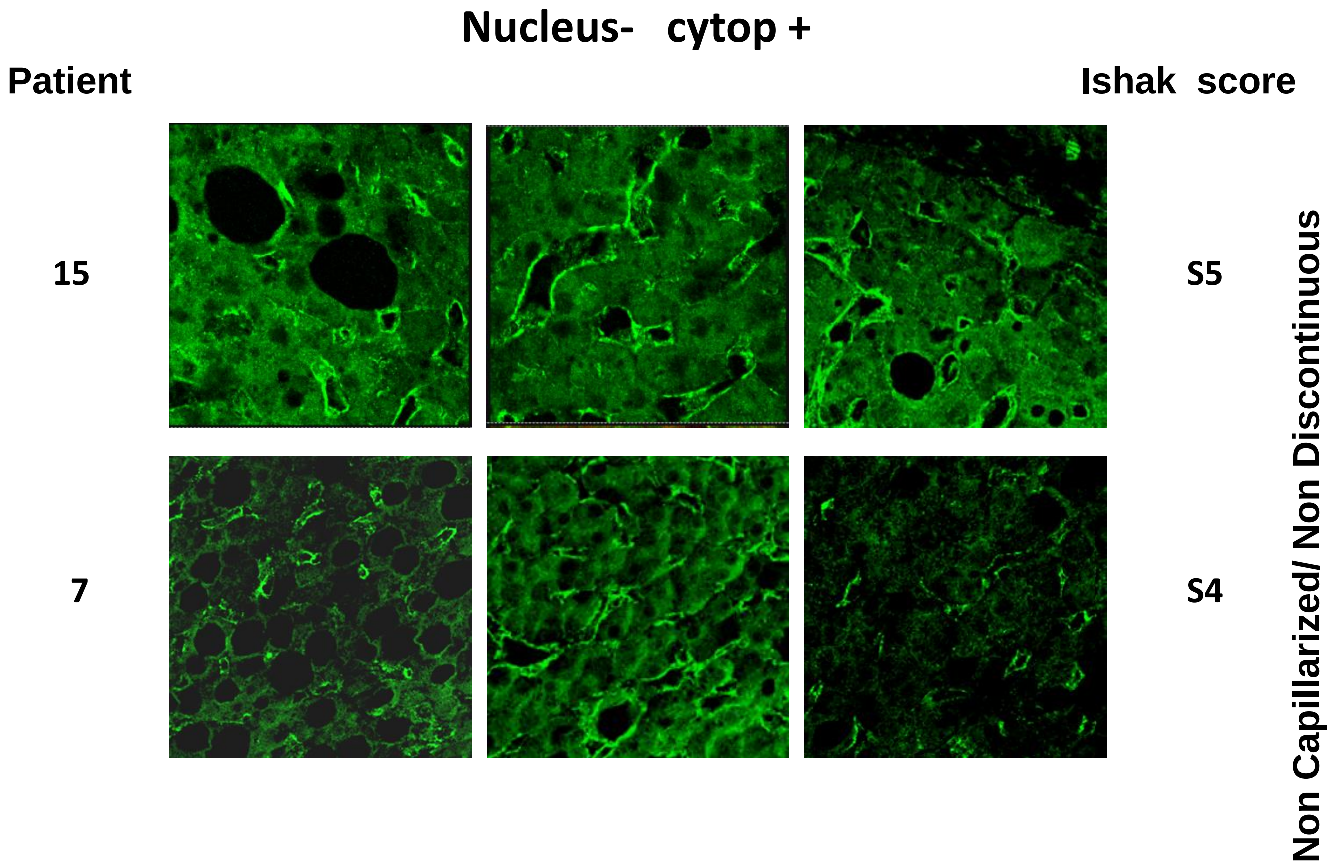

Confocal fuorescence microscopy images showing different pattern of Cav-1 distribution

Two patients for each staining pattern condition and 3 different areas from same patient are shown. Fibrosis score (according to Ishak grading) and LSECs changes are indicated on the left.

Supplementary Figure S3

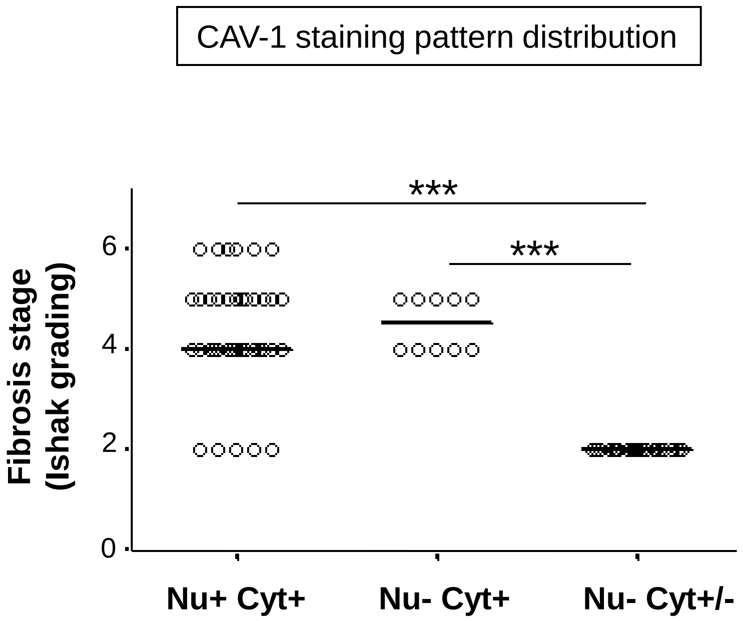

Dot plot analysis of Cav-1 staining pattern distribution in HCV-infected liver, according to the fibrosis stage. Individual dots correspond to the evaluation of immune-positivity distribution from randomly taken non-overlapping regions (n=5-6/ sample). Bars show the median. Significance was analysed using One-way ANOVA, Dunn's multiple comparison post hoc test. ***$P < 0.001$
